# The complete mitochondrial genome and phylogenetic analysis of *Haemaphysalis longicornis* Neumann (Acari: Ixodidae)

**DOI:** 10.1080/23802359.2017.1407707

**Published:** 2017-11-25

**Authors:** Jingjing Geng, Aihua Zheng, Zhen Zou, Xing Zhang

**Affiliations:** a College of Life Sciences, University of Chinese Academy of Sciences, Beijing, China;; b State Key Laboratory of Integrated Management of Pest Insects and Rodents, Institute of Zoology, Chinese Academy of Sciences, Beijing, China

**Keywords:** *Haemaphysalis longicornis*, mitogenome, phylogeny;tick

## Abstract

*Haemaphysalis longicornis* ticks are vectors or reservoirs of numerous infectious pathogens and cause a variety of human and animal diseases worldwide. However, there is limited knowledge on available genetic sequence. Herein, we extracted the complete mitochondrial genome (mitogenome) from enriched mitochondria of *H. longicornis* first time in ticks and gained its sequence with 14,718bp in length. The mitogenome consisted of 13 PCGs, 22 tRNA, 2 rRNA, and 2 noncoding regions. Also, the monophyletic phylogenetic position of *H. longicornis* is inferred based on 28 complete mitogenomes in total comprised of various species from Ixodida ticks in addition to the mitogenome of *H. longicornis*.

Ticks, the pathogen carriers of obligatory blood-sucking, are the second most important transmitters of various pathogens (Kiss et al. 2012; Liu et al. 2013). *Haemaphysalis longicornis* is the species with high medical and veterinary research importance, and has been reported to transmit *Babesia ovata* (Ohta et al. 1996), *B. gibsoni*, *Theileria* spp. (Piroplasmida: Theileriidae) (Fujisaki et al. 1994), Spotted Fever Group (SFG) *Rickettsia* spp. (Jongejan and Uilenberg 2004; Zou et al. 2011), and Severe fever with thrombocytopenia syndrome virus (SFTSV) which caused 10–15% mortality rate (Liu et al. 2014). Some properties of mitogenome, i.e. maternal inheritance, relatively high mutation rates, and the lack of recombination contribute to the broad consensus that mitogenome is widely regarded as genetic markers in molecular phylogenetic studies (Dermauw et al. 2009; Gu et al. 2014; Tao et al. 2014).

In this study, samples were the second-generation cultured ticks (specimen was deposited in the Institute of Zoology, Chinese Academy of Sciences, Beijing, China) from adult ticks collected in Qingzhou (36° 31′ 35″N, 118° 25′ 32″E), Shandong Province, China. Morphological characteristics and molecular methods of 16S rRNA amplifying and sequencing were used to identify the species. The live unfed adult ticks were collected and midguts were removed, and then used to enrich the mitochondria of *H. longicornis* with Qproteome^®^ Mitochondria Isolation kit. The mitochondrial DNA was extracted using the MasterPure DNA Purification kit (Epicentre Biotechnologies, Madison, WI). The mitogenome (GenBank accession no. MG450553) was 14,718 bp in length and consisted of 13 PCGs (*cox*1-3, *nd*1-6, *nd*4L, *cytb*, *atp*6, and *atp*8), 22 tRNA genes, 2 rRNA genes (16S rRNA and 12S rRNA), and 2 noncoding regions (*NCR1* and *NCR2*). The nucleic acid base content is 38.2% A, 13.1% C, 9.8% G, and 39.0% T. ATT start codon was used by n*d1, nd2, nd3, nd5, cox1*, and *cox2*. ATG codon was used by *atp6, nd4, nd4L* and *cytb*, and *atp8, cox3*, and *nd6* start at ATA. The most PCGs were terminated by TAA stop codon except *nd*3 and *cytb* that use TAG and *nd6* use the single T— as the stop codon.

The phylogenetic analysis (Figure 1) comprised 27 reported species mitogenome from GenBank and combined with *H. longicornis* mitogenome from this study. The phylogenetic tree was constructed using MEGA6 and utilized the Maximum Likelihood method, and the bootstrap test used 1000 replicates. The tree showed that the *H. longicornis* is located in the same clade with other *Haemaphysalis* genus species such as *H. flava*, *H. concinna*, *H. formosensis*, and *H. parva*, and displayed a closer phylogenetic relationship to *H. flava*, which indicated the monophyletic phylogenetic position of *H. longicornis*. The complete mitochondrial genome data would benefit to tick biogeography study and tick-borne diseases control.

**Figure 1.**
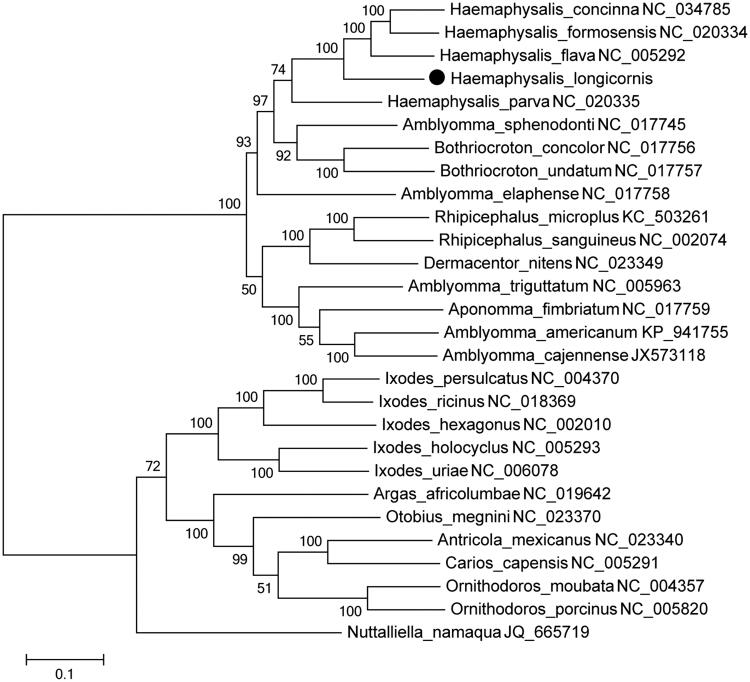
Phylogenetic relationship of Haemaphysalis longicornis among tick species was inferred based on mitogenome using the Maximum Likelihood method. The phylogenetic distances were computed using the Poisson correction method and were in the units of the number of amino acid substitutions per site. All ambiguous positions were removed for each sequence pair. The percentage of the bootstrap test in 1000 replicates was shown above the branches, and H. longicornis is labelled by “•”.
